# Customer satisfaction evaluation for drugs: A research based on online reviews and PROMETHEE-Ⅱ method

**DOI:** 10.1371/journal.pone.0283340

**Published:** 2023-06-22

**Authors:** Xiangqi Zhao, Lixiang Gao, Zhe Huang

**Affiliations:** 1 School of Business Administration, Shenyang Pharmaceutical University, Shenyang, Liaoning, China; 2 Institution of Regulatory Science for Medical Products, Shenyang Pharmaceutical University, Shenyang, China; CCET: Chandigarh College of Engineering and Technology, INDIA

## Abstract

Online reviews of consumers after purchasing drugs online reflect the factors affecting their satisfaction. How to understand customer satisfaction through online reviews and tapping their needs to improve satisfaction has become an urgent issue facing pharmaceutical e-commerce companies. Based on the online reviews of Alibaba Health Pharmacy, six representative OTC online medicines were selected for this study, including the following categories: tonics, anti-cold drugs, rheumatism and orthopaedic drugs, skin drugs, gastrointestinal drugs, vitamins, and calcium. By training and testing the LDA topic model, five potential topics are extracted as factors affecting customer satisfaction, including drug efficacy, drug cost performance, online customer service, logistics and transportation, and packaging. In this paper, Sentiment Analysis is used to process the review text to quantify the sentiment tendency of the review, and determine the evaluation scale value. Then, the random dominance among various drug factors is determined based on the Stochastic Dominance Rules. Finally, the PROMETHEE–Ⅱ method is used to determine the ranking value of the importance of each factor. The results suggest that the factors in different types of OTC drugs rank differently, which is also rationalized in this paper. This study provides a significant reference for improving customer satisfaction with pharmaceutical e-commerce.

## 1 Introduction

Online drug purchase refers to the drug transaction between consumers and e-commerce enterprises through the modern Internet. With the rapid period development of the Internet, an opportunity has been given to the joint operation between the drug retail industry and the Internet. Compared with offline physical stores, more people prefer buying commodities online. Due to the incentives of various websites, more consumers are willing to post online reviews after using products to express their preferences. However, these reviews help other consumers make purchase decisions [1–4]. With the popularization of information technology, online reviews have become easily accessible, and many scholars have used online reviews to analyze consumer preferences and decision-making behavior in e-shopping [5,6], tourism management [7,8], and hotel management [9,10]. Particularly, the global outbreak of the COVID-19 epidemic in 2020 has promoted the application of online health information and online health services, including telemedicine [11–13]. Besides, the Internet pharmaceutical e-commerce platform has received a rare development opportunity. Therefore, for pharmaceutical e-commerce, how to seize this opportunity, increase drug sales, enhance consumer satisfaction, and expand the number of users is worthy of attention. To achieve these goals, it is critical to extract and utilize the key information from online reviews. For example, according to these reviews, pharmaceutical e-commerce companies can clearly find out which aspect of a drug is more popular with consumers (such as drug cost performance, drug efficacy, logistics, and transportation, etc.). This can help e-commerce platforms remove a batch of low-quality drugs that are not popular. In this way, it can not only improve customer satisfaction but also contribute to the healthy development of the pharmaceutical e-commerce industry.

Of course, similar issues have also aroused eager discussions by many scholars. In recent years, many scholars have carried out research related to online reviews. Some scholars have conducted research on commodity levels or ranking methods based on online reviews. Najmi et al. proposed a commodity ranking method based on commodity online reviews and descriptions, they established a unified ranking for each commodity, thereby promoting the development of the online business industry [14]. Liu et al. proposed an online commodity evaluation method based on sentiment analysis technology and intuitionistic fuzzy set theory, ranking commodities through online reviews to facilitate consumers’ purchasing decisions [15]. Yang et al. proposed a method to integrate heterogeneous information, using textual sentiment and numerical scores to describe a specific commodity, resulting in the overall Electronic Word-of-Mouth (eWOM) score for each commodity and their ranking [16]. Based on online reviews, Li et al. used Social Network Analysis (SNA) theory to establish a comprehensive evaluation model, which was used to rank commodities and improve the insight of e-commerce companies on customer behavior [17]. Lin and Yu proposed a method of commodity selection based on prospect theory in online reviews and studied the bounded rationality of commodity levels and consumers [18]. Fan et al. proposed a commodities selection method based on online evaluation information and consumer expectations and conducted an empirical analysis based on the online evaluation information provided by the Autohome website for car selection [19]. Most of the above studies are based on online reviews to explore the ranking of commodities or make decisions for consumers, however, they do not categorize different review topics involved in online reviews; Moreover, they seldom consider consumers’ preferences for purchasing, and rarely involve their purchasing expectation.

In addition, some scholars have studied many important factors affecting it based on satisfaction. By studying 6402 online doctor reviews, Imbergamo et al. extracted factors that lead to dissatisfaction with joint surgeons, including clinical attitudes, adverse medical outcomes, and doctor proficiency [20]. Jung et al. checked employees’ job satisfaction factors through online reviews, and analyzed the importance of each factor from different perspectives such as team and time, Using strengths analysis and regression analysis to study the relationship between overall job satisfaction and some factors (such as organization, promotion opportunities, etc.) [21]. Based on the big data environment, Huang and Li used tools such as ICTCLAS and AntConc to mine hot reviews and studied the influencing factors of online pharmacy online reviews, providing a decision basis for online pharmacies to improve consumer trust and drug sales [22]. Guo et al. mined 266,544 online reviews from 25,670 hotels in 16 countries/regions, analyzed the factors that affect customer satisfaction, and calculated the relationship between each factor and customer gender or age. The relationship between five factors (such as hotel location, room size, etc.) and customer satisfaction was also studied by using the stepwise regression method [23]. Some of the above studies evaluate factors, or most of them use regression analysis and other models to explain the factors affecting satisfaction, however, there are still shortcomings and deficiencies, which are mainly described as follows: firstly, The independent variables of the regression model must have a strict correlation with the model itself, so strict assumptions must be made when modeling; secondly, the methods given by some studies are limited to the case where online review information is multi-level scoring, however, they do not consider online review information in text form. It is worth mentioning that the field of exploring online drug satisfaction evaluation based on online reviews is still in a blank state. Therefore, the research in this paper has strong significance.

With the continuous change in python, it is convenient to crawl online reviews efficiently. At the same time, many methods have emerged to deal with large and unstructured data such as online reviews. For example, Latent Dirichlet Allocation (LDA) is an unsupervised learning method that can effectively mine and discovers latent semantic topics in text data, which can identify several types of explicit topics in messy texts [24]. For online reviews, its topic can be viewed as a summary of customers’ feelings about a commodity or services. For example, based on the online reviews of a company review website in Korea, Jung and Suh et al. mined and extracted key factors affecting employee job satisfaction in IT, finance and other industries based on the LDA model, such as vacations, organizational culture, working hours, etc [25].

Sentiment analysis is a text mining analysis tool [26]. Based on the sentiment dictionary, words with different sentiment polarities can be mined from the text, thereby determining the sentiment polarity (positive, neutral, negative) of a certain text, which is convenient to quantify different emotions, establishing a quantitative evaluation scale value, and lay a good foundation for subsequent analysis and calculation. Therefore, this method is widely used in the evaluation of customer satisfaction in online reviews. For example, Srinivas and Rajendran extracted the factors that affect student satisfaction with the school, and counted the proportion of students’ positive attitudes, neutral attitudes and negative attitudes in each factor. Through the above methods, they have a comprehensive understanding of student satisfaction with each factor, and further study the situation of the university to give specific management opinions [27].

Stochastic Dominance Rules are decision rules that use partial information to form a partial order [28–30]. Its main feature is that it does not require too many strict assumptions, and can get more accurate alternative ranking results. This method is widely used in the fields of commodities, scheme selection, scheme optimization and service quality evaluation. It can determine the stochastic dominance relationship between any two factors, which is convenient for ranking the alternatives. The ranking methods mainly include ELECTRE–III method [29], Rough Set method [31], PROMETHEE–II method [32] and so on. Since the PROMETHEE-II method is a complete ordering method based on the priority relationship of levels, its mathematical properties are stable, and its ease of use and stability are strong [24,32]. Therefore, it is widely used in combination with the Stochastic Dominance Rules [33]. Thus, considering the massive online review information in the pharmaceutical e-commerce platform, this paper constructed a Stochastic Dominant Relationship Matrix between any two factors based on the review data of six categories of OTC drugs sold online. Considering that there is a large number of online reviews in the pharmaceutical e-commerce platform, this paper selected the reviews of six categories of OTC drugs sold online. Finally, a Stochastic Dominance Matrix of influencing factors affecting customer satisfaction in different drug categories is constructed using the Stochastic Dominance Rules, and the ranking of these factors in different drug categories is given by using the PROMETHEE–Ⅱ method. In this way, it can help pharmaceutical e-commerce companies to measure and evaluate customer satisfaction with online drug sales.

The remainder of this paper is organized as follows. The second part briefly introduces the method for topic extraction, sentiment analysis and factor ranking through online reviews. In the third part, a case study of Alibaba Health Pharmacy is presented to illustrate the use of the proposed method. Finally, discussions and conclusions are given in the fourth and fifth parts.

## 2 Method

### 2.1 Extraction of online review topics based on LDA model

Latent Dirichlet Allocation (LDA) is a probabilistic topic model proposed by Blei et al. in 2003. It is an unsupervised machine learning model that can be used to identify potential topic information in a corpus [34,35]. The model assumes that each word is extracted from a potential topic, which can be used by researchers to perform cluster analysis on different topics, as well as filter and classify different texts to achieve a systematic and organized effect.

**Fig 1:**
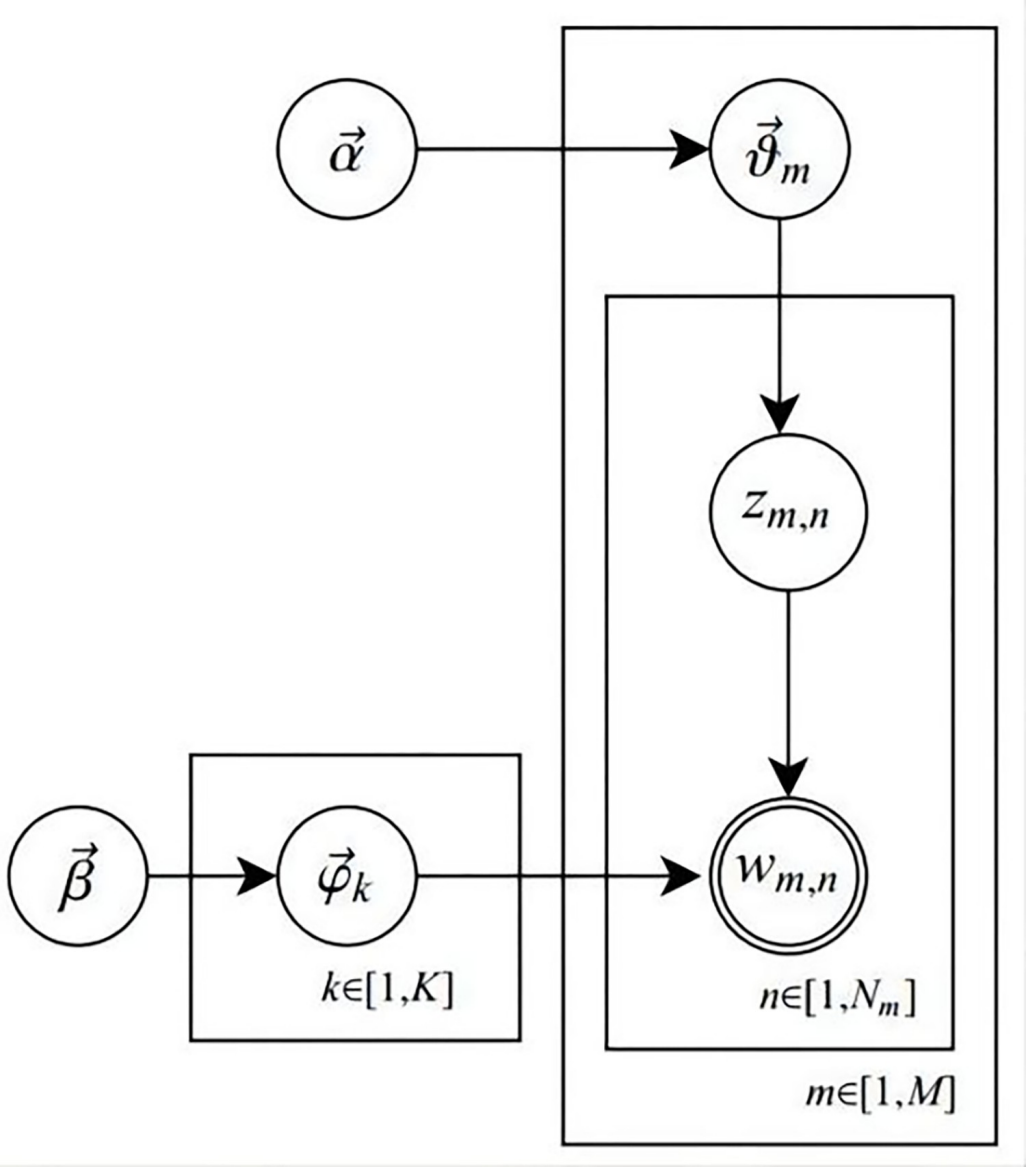
LDA model diagram for online review topic extraction.

This paper constructed a corresponding topic model based on customers’ online reviews of drugs sold online. We define an online review set as ***M***, which is used to represent all customer online reviews in pharmaceutical e-commerce. Let *m* denote each online review and assume it consists of *N* words. The set of words is represented by ***W***, where *w* = {*w*_1_, *w*_2_,…,*w*_*n*_}. Furthermore, assume that there are *K* implicit topics *z* in the set ***M***. Therefore, the generation process for each online review in the corpus is as follows: Firstly, for each review, a topic is drawn from the topic distribution; then extract a word from the word distribution corresponding to the extracted topic; repeat the above process until every word in the document is traversed. Therefore, the probability map of the LDA model extracted from the topic of online drug reviews for online sales is shown in Fig 1.

More clearly, the key steps are summarized as follows.

***Step 1***: Select *N* words, which obey the Poisson distribution with parameter *ξ*, that is, *N*~*Poisson*(*ξ*);

***Step 2***: Select the topic distribution of online reviews, satisfying ${\vec{\theta }}_{m}∼Dirichlet(\vec{\alpha })$, where *m* = {1,2,⋯,*M*}. So far, the word *w* has been formed. Since an online review consists of *N* words, the loop of *Step 3* needs to be executed:

***Step 3***: For each word *w* from the *N* words in the set ***W***, its corresponding topic *z*_*n*_ satisfies *z*_*n*_~*Multinomial*(*θ*). The polynomial probability conditioned on topic *z*_*n*_ is $P(w_{n}|z_{n},\vec{\beta })$.

In Fig 1, various symbols and their meanings are shown in Table 1.

**Table 1:** Symbol representation and definition in the LDA topic model based on Fig 1.

Symbol	Definition
*M*	the total number of online reviews, and *m* is a review, *m*∈[1,*M*]
*K*	the total number of topics, *k* represents a topic, *m*∈[1,*K*]
*N*_*m*_	the number of words in the *m*th review, and *n* represents the nth word, *n*∈[1,*N*_*m*_]
*w*_*m*,*n*_	the nth word of the *m*th review
*z*_*m*,*n*_	the nth topic of the *m*th review (hidden variable)
$\vec{\alpha }$	Dirichlet prior parameters for the multinomial distribution of a topic under the *m*th review
$\vec{\beta }$	Dirichlet prior parameters of the multinomial distribution of feature words under a topic
*θ*	the distribution of topics throughout the review
${\vec{\theta }}_{m}$	the topic distribution in the *m*th review
${\vec{\varphi }}_{k}$	the feature word distribution under the *k*th topic

As can be seen from Fig 1, to generate an online review, firstly, a topic distribution of the review must be generated, then a set of words of the corresponding topic must be generated; to generate a word, firstly, randomly select a topic based on the topic distribution of reviews, then randomly select a word based on the distribution of words in the topic. Repeat the above process until a complete review is generated.

Therefore, based on the basic equation of conditional probability, the joint posterior distribution probability of topic and feature words in online reviews is defined as

$$p(\vec{w},\vec{z}|\vec{\alpha },\vec{\beta })=p(\vec{w}|\vec{z},\vec{\beta })p(\vec{z}|\vec{\alpha })$$ (1)

It is easy to know that the marginal distribution of online reviews can be expressed as

$$p(w|\vec{\alpha },\vec{\beta })=\sum_{z_{n}}\int \prod_{n=1}^{N}p(z_{n}|\theta )p(w_{n}|z_{n},\vec{\beta })p(\theta |\vec{\alpha })d\theta$$ (2)

To sum up, the topic model of online reviews for drugs sold online using the LDA model is as follows:

Assuming that each word *w*_*m*,*n*_ in a review is given. Next, the topic *z*_*m*,*n*_ of each word should be calculated, as well as the posterior probability distribution of the topic distribution ${\vec{\theta }}_{m}$ of each review and the probability distribution ${\vec{\varphi }}_{k}$ of the words within each topic. Since it is very difficult to directly obtain the distribution probability of hidden variables, this paper adopts the Gibbs sampling algorithm, approximate reasoning is used to determine the parameters of the topic model, and boundary integrals are performed on them to facilitate statistical inference of hidden variables, thereby obtaining the parameter distribution [36]. The statistical principles involved are no longer discussed and proved in this paper.

This paper uses Python to run and debug the code to implement an LDA-based extraction model for online drug review topics. The first step is to determine the total number of online review topics *K* of drugs sold online according to a reasonable test, and dig out the meaning of the topic according to each topic. The second step is to take each online review topic as a factor affecting customer satisfaction, denoted as *F*_*k*_ and satisfying *k*∈[1, *K*], *k*∈***Z***^**+**^. The third step is to determine the topic of each online review.

### 2.2 Text sentiment analysis for drugs

**Fig 2:**
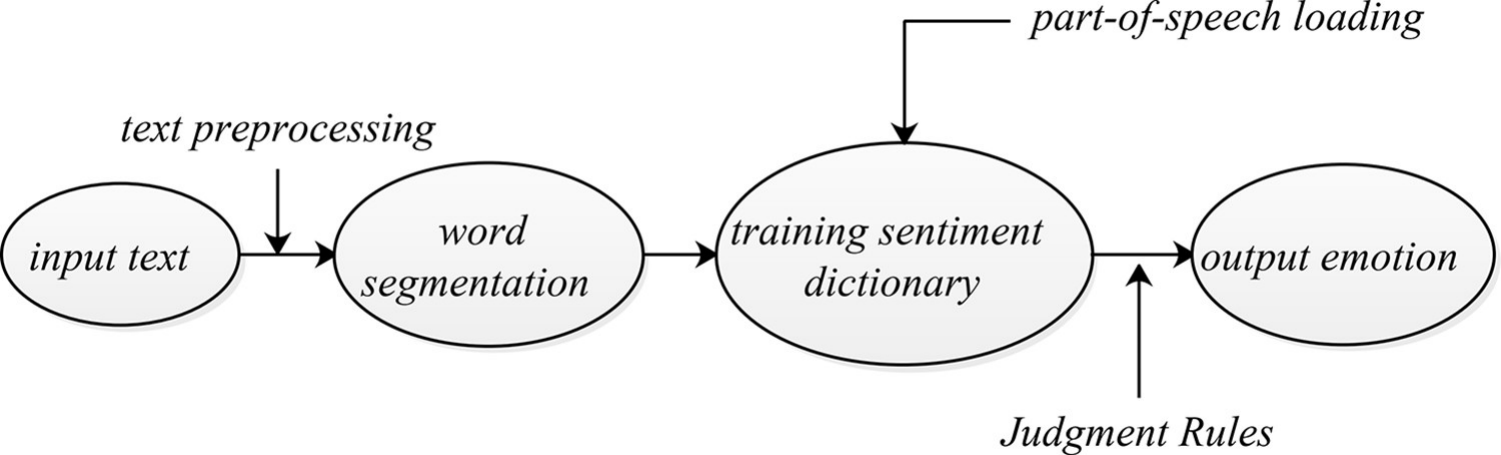
Text sentiment analysis process based on sentiment dictionary.

Text sentiment analysis, also known as opinion mining, refers to the analysis of subjective texts with emotional color to mine the emotional tendencies contained in them. Its methods can be divided into three categories: sentiment dictionary-based analysis methods, traditional Machine Learning-based methods, and Deep Learning-based methods [37]. Since each online review text is relatively short and has a small number of words, this paper adopts a dictionary-based text sentiment analysis method. The general process of this method is shown in Fig 2.

The process shown in Fig 2 can be briefly described as follows. Firstly, input the text, and preprocess the data by denoising and erasing invalid characters. Then the word segmentation operation should be carried out, and various words of different degrees in the emotional dictionary should be put into the model for training. Finally, the emotion type is output with the help of emotion judgment rules. More clearly, the text sentiment analysis steps for online reviews of drugs sold online are summarized as follows.

***Step 1***: Build a sentiment dictionary. The sentiment dictionaries used in this study are *HowNet* (Chinese sentiment dictionary) and National Taiwan University Simplified Chinese sentiment polarity dictionary. In order to maximize the coverage of word emotion, some popular Internet terms are additionally introduced to ensure that the text is updated with the times [38]. In this paper, the symbols $V_{C}^{+}$, $V_{C}^{0}$ and $V_{C}^{+}$ are used to denote derogatory, neutral and positive sentiment words, which all belong to the set ***V***_***C***_. Some specific emotional words will not be given here, the relevant contents will be given later.

***Step 2***: Determine the polarity of sentiment words. If some words cannot find the corresponding sentiment words in the sentiment dictionary, the polarity should be determined manually and then stored in the sentiment dictionary, and *Step 1* must be backtracked to update the sentiment dictionary synchronously. Otherwise, go to *Step 3*. Let *Emo*_*m*,*i*_ denote the *i*th sentiment word of online review *m*, where *i*∈[1,2…,*I*]. The polarity of the sentiment word *Emo*_*m*,*i*_ is represented by *Polar*(*Emo*_*m*,*i*_), which is defined as

$$Polar(Emo_{m,i}|V_{C})=\{\begin{array}{cc} -1, & Emo_{m,i}\in V_{C}^{-}\\ 0, & Emo_{m,i}\in V_{C}^{0}\\ 1, & Emo_{m,i}\in V_{C}^{+} \end{array}$$ (3)

***Step 3***: Deal with degree adverbs. Online reviews published by consumers generally include some degree adverbs used to deepen the tone to enhance the expressive intensity of subjective feelings, like these words: too, very, extremely, etc. Based on *HowNet* and National Taiwan University Simplified Chinese Emotional Polarity Dictionary, this paper refers to commonly used adverbs of degree as evaluation grades, and the degree values are defined as 2 and 1, respectively. The degree adverb level of the *i*th sentiment word that modifies the *m*th review is denoted by *Deg*(*Emo*_*m*,*i*_), and some examples of rules are shown in Table 2.

**Table 2:** Sentiment word degree adverbs and their corresponding grades in online reviews.

*Deg(Emo*_*m*,*i*_ *)*	degree adverb
2	extremely, pretty, quite, very, much, too, greatly, almost, etc.
1	little, slightly, barely, passably, etc.

***Step 4***: Deal with negative adverbs. Negative adverbs appear in some reviews, which also require special attention. Obviously, for a review, if there are an odd number of negative adverbs, the polarity of the review will be changed; if there are an even number of negative adverbs, the polarity will not be changed. In this paper, we assume that the number of negative adverbs in an online review is *H*. Usually, negative adverbs appear at most twice in a review, so *H* = 1, 2. Therefore, the sentiment analysis score of the *m*th reviews is defined as

$$\begin{array}{l} Score_{m}=\sum_{i=1}^{I}{\left[{\left(-1\right)}_{m,i}\right]}^{H}Polar(Emo_{m,i}|V_{C})\times Deg(Emo_{m,i})\\ s.t.\;H=1,2;Deg(Emo_{m,i})=1,2 \end{array}$$ (4)

When *Step 3* is completed, the evaluation criteria of customer satisfaction can be further obtained by using the sentiment analysis score. In this paper, the set ***S*** is used to represent the evaluation standard of customer satisfaction, which satisfies *S* = {*S*_1_, *S*_2_, *S*_3_}. Let *S*_*e*_ represent the *e*th evaluation scale, then

$$S_{e}=\{\begin{array}{cc} 3, & e=3;\;Score_{m}\in (0,+\infty )\\ 2, & e=2;\;Score_{m}=0\\ 1, & e=1;\;Score_{m}\in (-\infty ,0) \end{array}$$ (5)

In Eq (5), *S*_3_, *S*_2_, and *S*_1_ are used to express satisfaction, general and dissatisfaction, respectively.

### 2.3 Ranking of influencing factors

In the above, the factors affecting customer satisfaction in online reviews have been obtained through the LDA model, and the evaluation scale value of customer satisfaction has also been determined. This part will use Stochastic Dominance Rules to calculate the cumulative distribution function and expectation of the evaluation scale in each influencing factor. Based on the above, a Stochastic Dominant Relationship Matrix will be established. And based on the priority function of the PROMETHEE-II method, the “*outflow*” value, “*inflow*” value and ranking value of each factor corresponding to each type of drug are calculated.

According to the characteristics of drugs, *s* categories of drugs sold online sale are determined. Denote the set composed of class s drugs as set ***C***, and *C* = {*C*_1_,*C*_2_,⋯,*C*_*i*_,⋯,*C*_*s*_}. According to the *K* medical e-commerce online review topics, the set of factors influencing customer satisfaction of online reviews is set as ***F***, and *F* ={*F*_1_,*F*_2_,⋯,*F*_*k*_,⋯,*F*_*K*_}. According to the customer satisfaction analysis based on the LDA topic model and Sentiment Analysis, the influencing factors of online reviews *m* and their satisfaction evaluation scale are obtained.

For online review set ***D***, count the number of reviews whose influence factor is *F*_*k*_ in online drug category *C*_*i*_, and denote it as *ψ*_*ik*_; For the online reviews of the drug category *C*_*i*_, count the number of reviews whose influencing factor is *F*_*k*_ and the satisfaction value is *S*_*e*_, then denote it as $\psi_{ik}^{e}$. Therefore, the probability of *S*_*e*_ can be defined as

$$\begin{array}{l} P{\left(dos\right)}_{ik}^{e}=\frac{\psi_{ik}^{e}}{\psi_{ik}}\\ s.t.\;i=1,2,\cdots ,s;\;k=1,2,\cdots ,K;\;e=1,2,3 \end{array}$$ (6)

Then the following Eqs (7) and (8) can be obtained, which are as follows:

$$F(t)=\sum_{t}P{\left(dos\right)}_{ik}^{e}=\sum_{t}\frac{\psi_{ik}^{e}}{\psi_{ik}}\;s.t.\;t\geq S_{e}$$ (7)

$${\vec{E}}_{i}={\left(\gamma_{ik}\right)}_{K\times 1}={\left[\sum_{e=1}^{3}P{\left(dos\right)}_{ik}^{e}S_{e}\right]}_{K\times 1}$$ (8)

Eq (7) is the cumulative distribution function of the factor *F*_*k*_ in the category *C*_*i*_ whose satisfaction is *S*_*e*_. Eq (8) represents the expected vector of the evaluation scale with the factor *F*_*k*_ in the category *C*_*i*_. Since there are *K* influencing factors, the vector is a column vector with *K* rows and one column.

Then, a Stochastic Dominant Matrix needs to be established [28]. Specifically, this paper assumes that *G*_*ik*_(*t*) represents the cumulative distribution function of the influencing factor *F*_*k*_ in the drug category *C*_*i*_, *G*_*ip*_(*t*) represents the cumulative distribution function of the influencing factor *F*_*p*_ in the drug category *C*_*i*_. The Stochastic Dominant Relationship Matrix ${\left(R_{kp}^{i}\right)}_{K\times K}$ between two factors is constructed as follows:

$$R_{kp}^{i}=\{\begin{array}{cc} FSD, & G_{ik}(t)FSDG_{ip}(t)\\ SSD, & G_{ik}(t)SSDG_{ip}(t)\\ TSD, & G_{ik}(t)TSDG_{ip}(t)\\ -, & others \end{array}\;s.t.\{\begin{array}{c} k,\;p=1,2,\cdots ,K\\ i=1,2,\cdots ,m \end{array}$$ (9)

In Eq (9), when *G*_*ik*_(*t*) randomly dominates *G*_*ip*_(*t*), then the factor *F*_*k*_ randomly dominates factor *F*_*p*_. Among them, *FSD*, *SSD*, and *TSD* represent first-order dominance, second-order dominance, and third-order dominance, respectively [30]. Briefly describe Stochastic Dominance Rules as follows:

Assuming that the random variables *X* and *Y* are both defined on the interval [*a*,*b*], their distribution functions are *F*(*x*) and *G*(*x*) respectively, and *F*(*x*)≠*G*(*x*). If

$$\forall x\in [a,b],\;H_{1}(x)=F(x)-G(x)\leq 0$$ (10)

then *F*(*x*) first-order random occupation is better than *G*(*x*), denoted as *F*(*x*)*FSDG*(*x*). If

$$\forall x\in [a,b],\;H_{2}(x)=\int_{-\infty }^{x}\left[F(t)‐G(t)\right]dt\leq 0$$ (11)

then *F*(*x*) second-order random occupation is better than *G*(*x*), denoted as *F*(*x*)*SSDG*(*x*). If

$$\forall x\in [a,b],\;H_{3}(x)=\int_{-\infty }^{x}\int_{-\infty }^{u}\left[F(t)-G(t)\right]dtdu\leq 0$$ (12)

then *F*(*x*) third-order random occupation is better than *G*(*x*), denoted as *F*(*x*)*TSDG*(*x*).

Next, based on the Dominant Relationship Matrix and the PROMETHEE-II method, this paper will determine the *K*-order Dominant Matrix between the factors *F*_*k*_ and *F*_*p*_ in the category *C*_*i*_, the matrix is denoted as $RD_{i}={\left[rd_{i}(F_{k},F_{p})\right]}_{K\times K}$. Each element in the matrix should satisfy

$$rd_{i}(F_{k},F_{p})=\{\begin{array}{cc} 1 & F_{k}SD*F_{p};\gamma_{ik}\in [\gamma_{ip}+\varepsilon_{i},+\infty ]\\ \frac{\gamma_{ik}-\gamma_{ip}}{\varepsilon_{i}} & F_{k}SD*F_{p};\gamma_{ik}\in [\gamma_{ip},\gamma_{ip}+\varepsilon_{i}]\\ 0 & others \end{array}$$ (13)

$$\varepsilon_{i}=\frac{2}{K(K-1)}\sum_{k=1}^{K}\sum_{p=1}^{K}\left(\gamma_{ik}-\gamma_{ip}\right)\;s.t.\{\begin{array}{l} \gamma_{ik}\geq \gamma_{ip}\\ k,\;p=1,2,\cdots ,K;\;k\neq p\\ i=1,2,\cdots ,s \end{array}$$ (14)

In Eq (13), SD* is expressed as first-order dominance or second-order dominance or third-order dominance. It is easy to know that the *rd*_*i*_∈[0,1], and when the value of *rd*_*i*_ increases, the degree of satisfaction of the factor *F*_*k*_ is more obvious than that of *F*_*p*_. In Eq (14), *ε*_*i*_ is the customer’s preference threshold for *F*_*i*_ [18,36], which is related to the expected difference between the two factors.

According to the Stochastic Dominance Matrix obtained above, this part calculates the credibility that a satisfaction factor is superior to the other factor, that is, the “*outflow*” value and “*inflow*” value of a certain influencing factor in the drug category *C*_*i*_. Φ^+^(*F*_*k*_) is the “*outflow*” value, which indicates the credibility of the factor *F*_*k*_ being superior to other factors, as the value increases, the reliability increases accordingly; Φ^−^(*F*_*k*_) is the “*inflow*” value, indicating the reliability of the factor being inferior to the other factors, as the value decreases, the confidence decreases accordingly.

Taking the factor *F*_*k*_ as an example, the calculation equations of its “*outflow*” value and “*inflow*” value are as follows:

$$\Phi_{k}^{+}(F_{k})=\sum_{k=1}^{K}rd_{i}(F_{k},F_{p})$$ (15)

$$\Phi_{k}^{-}(F_{k})=\sum_{k=1}^{K}rd_{i}(F_{p},F_{k})$$ (16)

The constraints are the same as above, and *k*≠*p* must be satisfied. On the basis of Eqs (15) and (16), the net flow of the calculation factor *F*_*k*_ is

$$\Phi_{k}=\Phi_{k}^{+}(F_{k})-\Phi_{k}^{-}(F_{k})$$ (17)

According to Φ_*k*_, the ranking of each factor can be calculated. As Φ_*k*_ increases, the importance of the factor increases accordingly.

## 3 Empirical analysis and results

**Fig 3:**
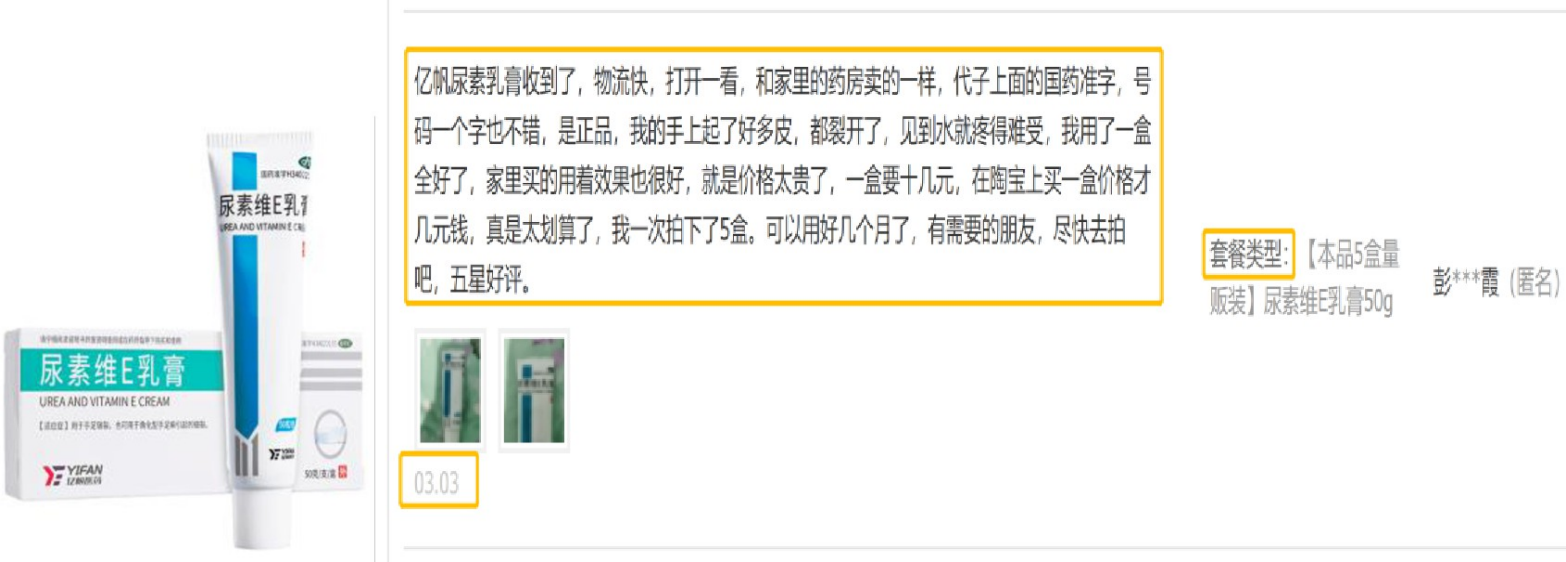
An example of the collected data (Taking *Urea and Vitamin E Cream* as an example, the left side of the figure shows the shape and packaging of the drug, and the right side of the figure shows one of its online reviews). Source: Ali Health Pharmacy.

In this paper, the case study is conducted with respect to Ali Health Pharmacy (https://www.alihealth.cn/), an online pharmacy that is very popular in China. The related data is collected from the Official website of Ali health pharmacy (https://www.liangxinyao.com/). According to the specific sales situation, we selected six representative OTC drugs. Fig 3 shows an example of the collected data. It can be seen from Fig 3 that the collected data include the customer’s review, the customer’s rating and the date. A total number of 50,535 online reviews were collected by June 2022. We screened these data mainly in three ways. Firstly, we looked at a number of comments and eliminated those with fewer than 15 Chinese characters, as they were not linguistically rich and did not facilitate the extraction of diverse and valid information from them. Secondly, we eliminated some low-quality online reviews (with multiple repetitive characters in a row), such as “very good, very good, very good, very good, very good, very good, very good, very good, very good, very good, very good…”. There are a lot of words in these comments, but only expresses a single and unclear message, which is not in line with the richness of online comments and cannot be used to extract useful topics. Thirdly, some consumers post online reviews that are not related to the drug. These online reviews appear to have been copied from elsewhere and contain a large amount of irrelevant text, which is wordy and not easily detected and needs to be manually checked and eliminated.

After removing the invalid reviews, we obtained 37,393 valid online reviews. These data are processed by *jieba* word segmentation, and the result of word segmentation is stored in a new document, which retains necessary words and removes stop words. The related information of the collected reviews is given in Table 3.

**Table 3:** Online reviews distribution over Different categories of drugs.

Drug Category	Category name	Total number of reviews
*C*_1_	Tonics	7352
*C*_2_	Anti-cold drugs	5526
*C*_3_	Rheumatism and orthopaedic drugs	4292
*C*_4_	Skin drugs	11028
*C*_5_	Gastrointestinal drugs	4779
*C*_6_	Vitamins and calcium	4416

### 3.1 Topics in online reviews

This section uses the LDA model to determine the optimal number of topics *K* for reviews. The optimal number of topics is generally determined by perplexity [39]. As the perplexity decreases, the model performance will be better. Topic-coherence is another major model for optimal topic number selection [40–43]. However, domestic studies rarely use this method to determine the number of topics. As one of the important techniques for estimating the number of topics, topic coherence is the most effective method to measure the quality of topics. As the coherence increases, the model performance will be better. Some theoretical knowledge of topic perplexity and topic coherence involved will not be repeated here.

**Fig 4:**
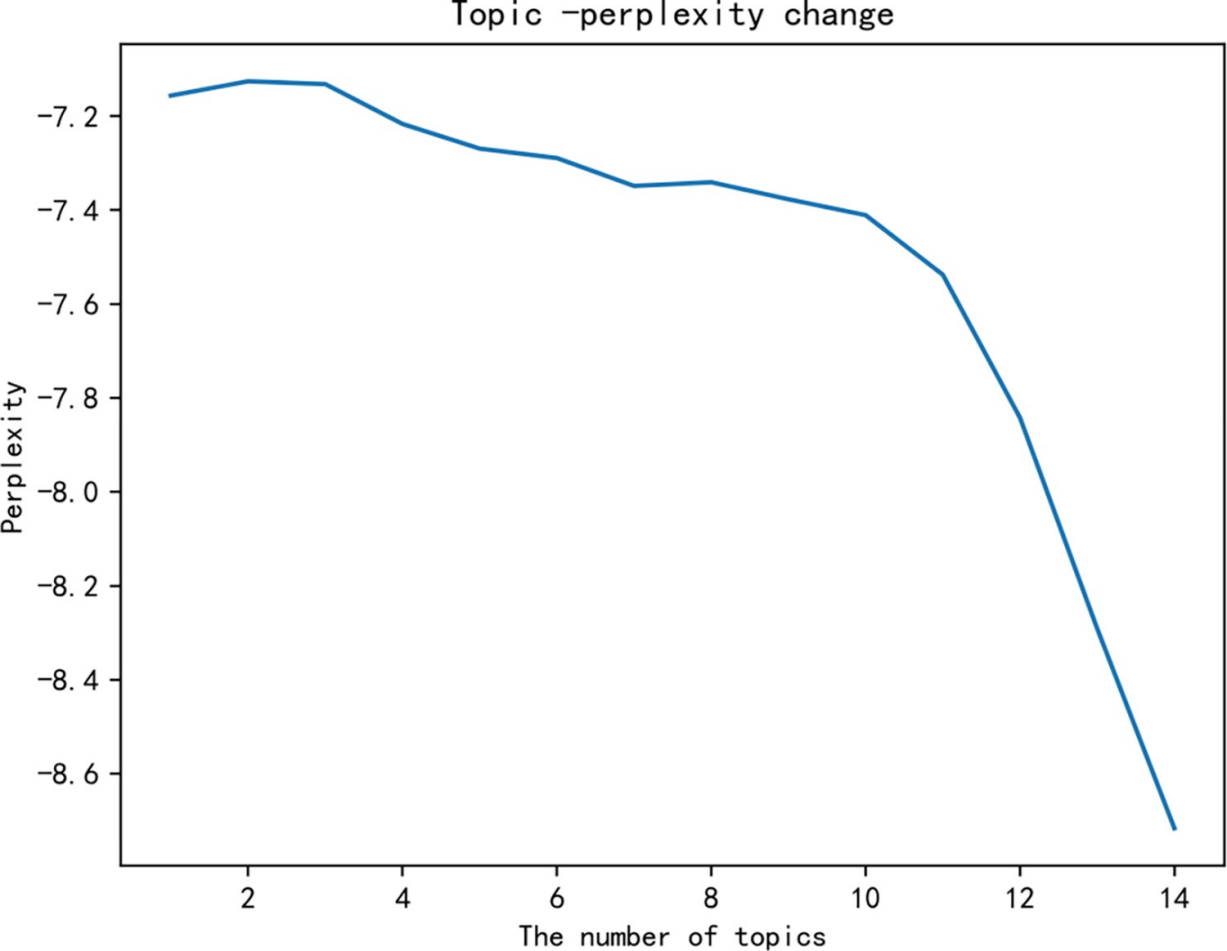
Topic-confusion perplexity graph.

Using scientific methods to determine the optimal number of topics *K* is particularly critical for the development of follow-up research. This paper will use a combination of perplexity and topic coherence to determine the *K* value. Through 15 model tests in this study, the topic-perplexity image is obtained as shown in Fig 4.

**Fig 5:**
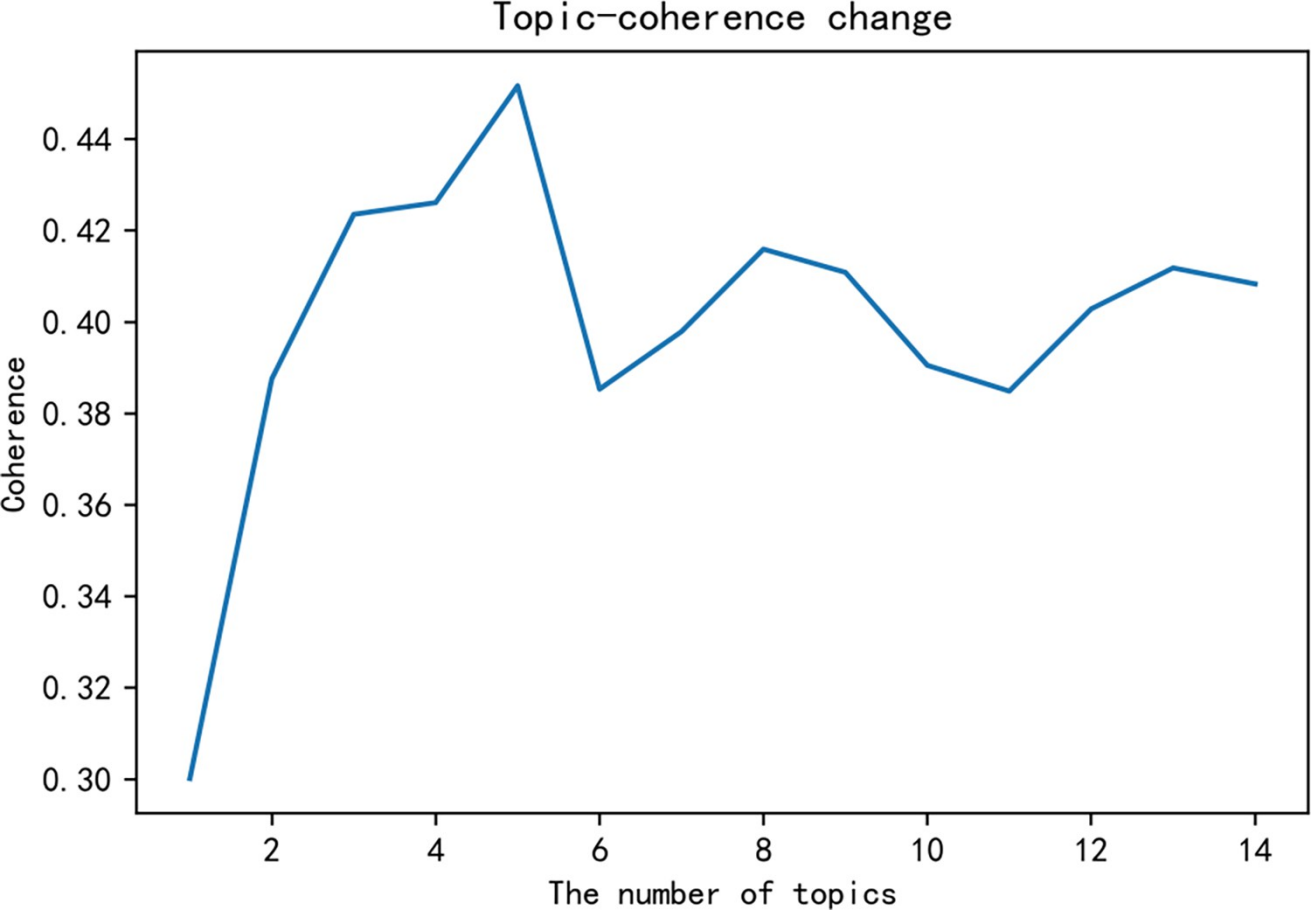
Topic-coherence perplexity graph.

It can be seen from Fig 4 that when *K*∈[3,7]∪[8,+∞], the perplexity of the topic gradually decreases. Obviously, when *K*∈[10,+∞], the LDA model is overfitted. Therefore, the range of the optimal number of topics *K* falls in the interval [3,7]. By further testing, a topic-coherence graph is obtained, which is shown in Fig 5.

**Fig 6:**
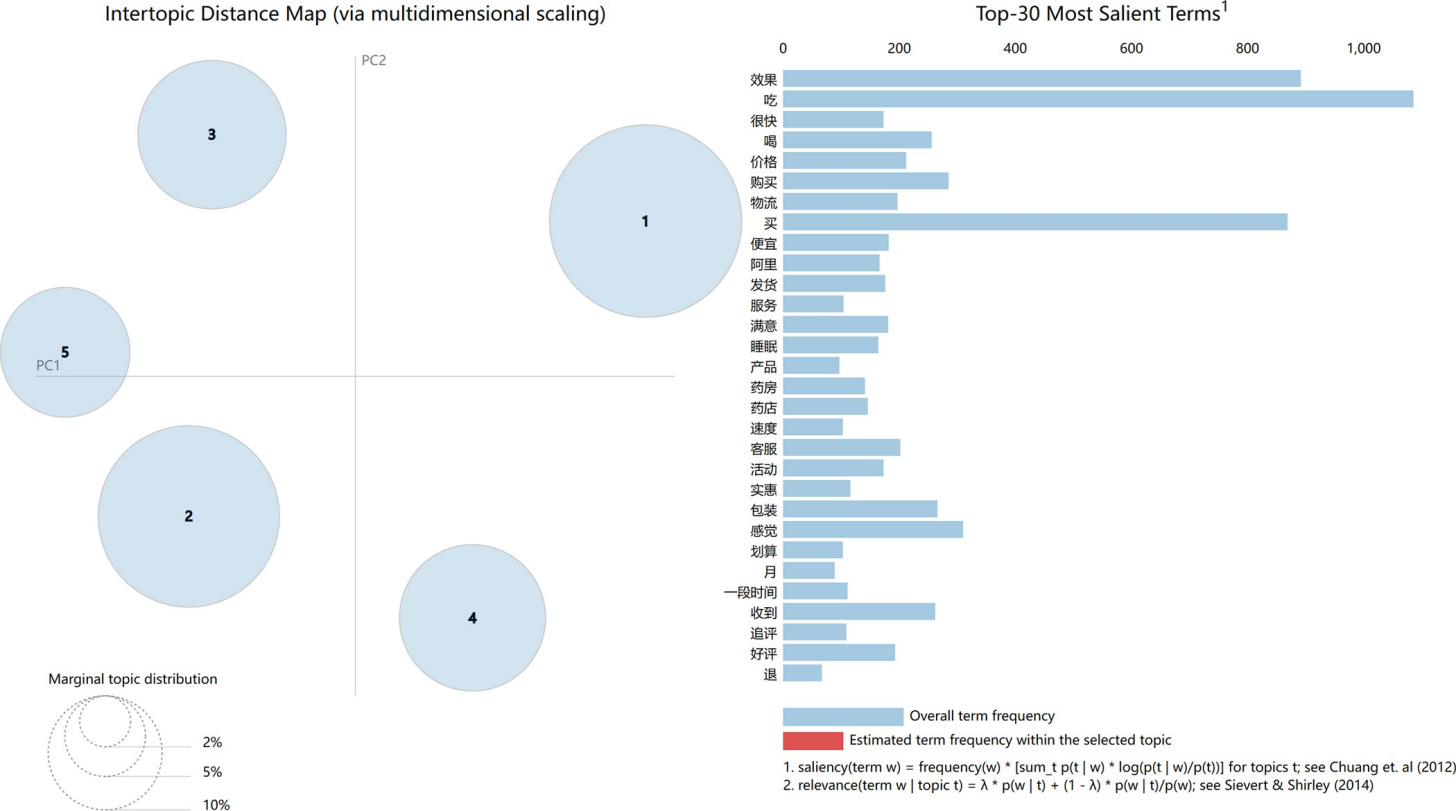
LDA visualization analysis based on *pyLDAvis*.

Fig 5 shows that when the value of *K* is five, the image has the largest peak in the given interval. When K>5, the curve showed a sharp downward trend, and the trend did not reach the previous peak. Since “*K* = 5” satisfies the interval [3,7] given by the *K* value. Based on the above analysis, the optimal number of topics can be determined to be five. In order to verify its rationality, the number of topics in this study is set to five, and the *pyLDAvis* tool is used for LDA visualization analysis. The relevant situation is shown in Fig 6.

In Fig 6, the two axes are meaningless because the semantic space is high-dimensional and it simply gives a representation of each topic on the axes. The size of the circles indicates how often the topics occur, and the position between the circles indicates how close the topics are to each other. If there is an overlap between the circles, then the topics contain feature words that cross over. The five topics are shown on the axes on the left side of the figure, and the 30 words that appear more frequently throughout the text are shown on the right side of the figure. In general, if the circles can be perfectly separated, the classification effect of the subject will be the best. If the overlap between the circles is high, there are more intersecting words between the two topics and the topic classification will not work well. It can be seen from Fig 6 that the independence of the fifth topic is quite good, and the difference in the size of each circle is extremely small, whose positions are evenly distributed in each quadrant. Therefore, the model works extremely well, and the optimal number that *K* = 5 is reasonable.

**Fig 7:**
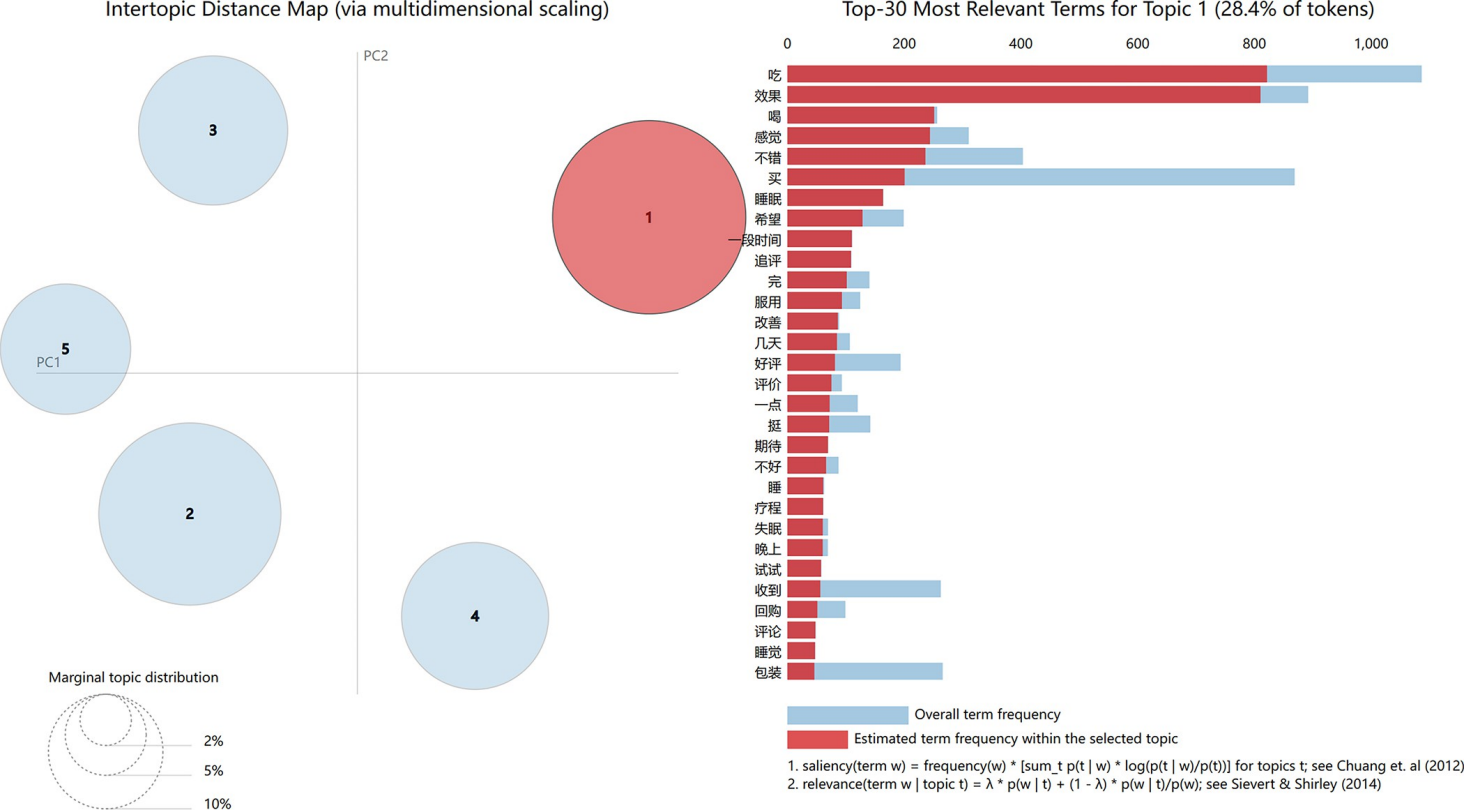
LDA visualization analysis diagram based on *pyLDAvis*—taking topic 1 as an example.

Fig 7 shows the relevant information, which uses the topic as an example. The circle area numbered 1 on the left side of the figure is marked in red, indicating that topic 1 is selected in the visual analysis. The top 30 weighted words contained in topic 1 are displayed on the right side of the figure, which shows the proportion of this word in the total text.

According to the top 20 words ranked by probability under each topic and the logical relationship between these words, the name corresponding to each topic was determined. The names of topics 1–5 are as follows: drug efficacy (*F*_1_), drug cost performance (*F*_2_), online customer service (*F*_3_), logistics and transportation (*F*_4_), drug packaging (*F*_5_), these five topics were considered as factors affecting customer satisfaction in six categories of OTC drugs sold online.

Taking Topic 1 and Topic 2 as examples, the two topics and the keywords they contain are shown in detail. Topic 1 is called drug efficacy (*F*_1_), and its keywords include words such as effect, good, feeling, improvement, etc.; topic 2 is called drug cost performance (*F*_2_), which mainly includes price, cheap, pharmacy, affordable, etc. Their details are shown in Table 4.

**Table 4:** Keywords and their probability values of drug efficacy (*F*_1_) and drug cost performance (*F*_2_).

Topic 1: Drug Efficacy (*F*_1_)		Topic 2: Drug Cost Performance (*F*_2_)	
Included keywords	Probability of keywords	Included keywords	Probability of keywords
吃(eat)	0.0821	买(buy)	0.0527
效果(effect)	0.0819	价格(price)	0.0243
喝(drink)	0.0253	便宜(cheap)	0.0205
感觉(feel)	0.0244	阿里(Ali)	0.0192
不错(good)	0.0241	活动(activity)	0.0177
买(buy)	0.0206	药店(pharmacy)	0.0163
睡眠(sleep)	0.0167	药房(pharmacy)	0.0166
希望(hope)	0.0131	实惠(affordable)	0.0134
一段时间(a period of time)	0.0117	划算(Cost-effective)	0.0129
追评(follow up review)	0.0112	正品(genuine)	0.0112
完(finish)	0.0107	购买(buy)	0.0104
服用(take)	0.0094	客服(customer service)	0.0106
改善(improve)	0.0091	快递(express)	0.0102
几天(several days)	0.0082	说(say)	0.0104
好评(praise)	0.0086	值得(deserve)	0.0089
评价(evaluation)	0.0083	优惠(discount)	0.0085
一点(a little)	0.0071	信赖(trusted)	0.0087
挺(quite)	0.0075	东西(thing)	0.0077
期待(expect)	0.0079	实体店(Physical store)	0.0073
不好(bad)	0.0071	不错(good)	0.0079

### 3.2 Ranking of factors affecting customer satisfaction

Sentiment Analysis of text is performed based on the extracted online review data. The sentiment dictionaries referenced in this part are *HowNet* and National Taiwan University Simplified Chinese Sentiment Polarity Dictionary. In addition, the actual experience of the consumer with the drug is also considered, and some additional words that are more relevant to the drug experience are added to ensure broad coverage of emotional words. The polarity of the sentiment dictionary is divided into three categories: positive, neutral and negative. The sentiment words of different polarities and their related examples are shown in Table 5. As a result, a customer satisfaction sentiment dictionary based on online sales of OTC drugs is constructed.

**Table 5:** Emotional words and examples of different polarities.

The polarity of sentiment dictionary	Examples of some emotional words
Positive (+)	退烧(bring down a fever), 痊愈(heal), 消肿(reduce swelling), 恢复(recover), 有效(effective), yyds (i.e., an internet term for compliments), 棒(excellent), 消炎(anti-inflammatory), 卫生(sanitary), 信赖(trusted), 给力(awesome), 显著(significant), 奥利给(an internet term for compliments, i.e. awesome), 舒畅(comfortable), 实惠(affordable), 安心(assured), 细腻(delicate), 超值(Cost-effective), 划算(Cost-effective), 热情(enthusiastic), 完美(perfect), 便宜(cheap), 严实(tight), 备用(spare), 好评(praise)
Neutral (0)	不疼(painless), 正常(normal), 可以(ok), 普通(ordinary), 一般(general)
Negative (-)	差评(bad review), 假药(fake), 苦(bitter), 难受(uncomfortable), 恶心(disgusting), 加重(worsening symptoms), 慢(slow), 莫名其妙(inexplicable), 差(bad), 讨厌(dislike), 无语(speechless), 瘪(shrunk), 变形(deformed), 垃圾(rubbish), 碎(broken), 糟糕(terrible), emo(emotional), 烂(rotten), 破损(broken), 坑(nasty), 失望(disappointed), 敷衍(perfunctory)

**Fig 8:**
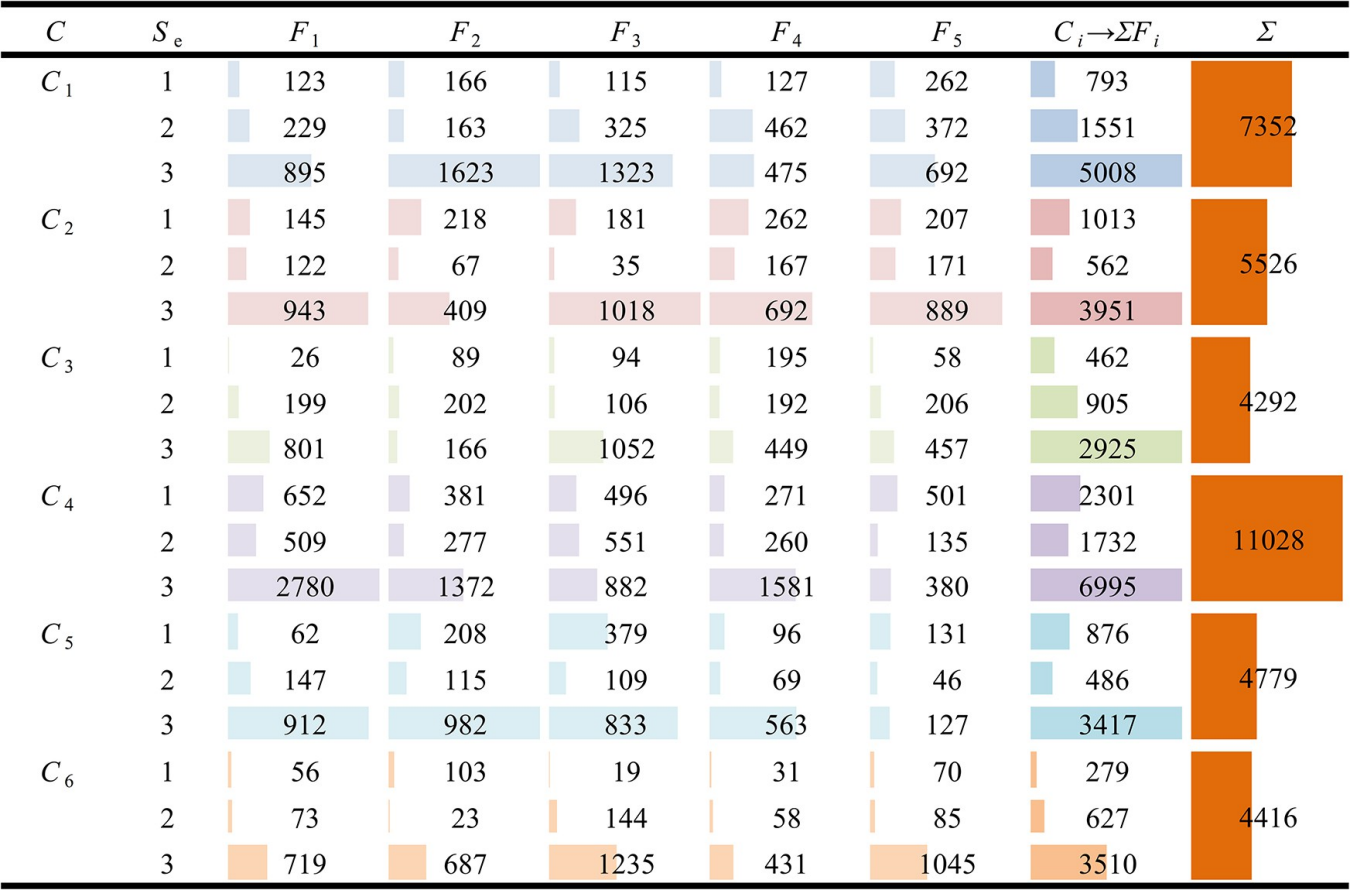
The number of reviews of different types of OTC drugs under various influencing factors and different satisfaction scales.

After constructing the sentiment dictionary, the sentiment polarity of words in online reviews can be determined by Eq (3). Eq (4) can be used to determine the Sentiment Analysis score of an online review of drugs. Finally, the sentiment score of the entire review can be judged by Eq (5), and the value of *S*_*e*_ can be determined, that is, the satisfaction degree of consumers. For each drug category, the number of reviews of consumer satisfaction under different factors has been determined, which is shown in Fig 8.

**Fig 9:**
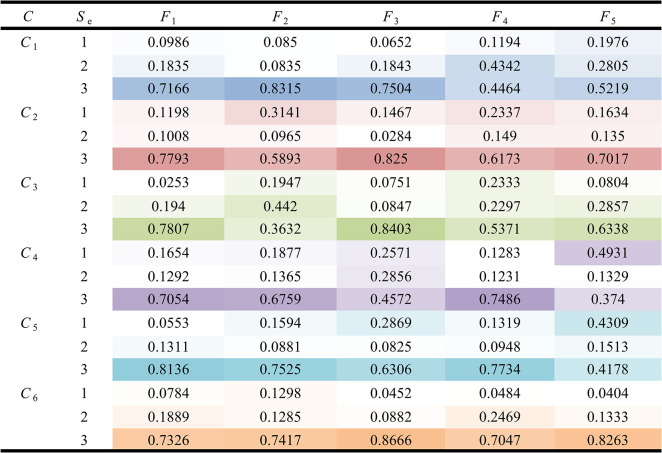
Probability distribution of reviews for different categories of OTC drugs under various influencing factors and different satisfaction scales.

On the basis of Fig 8, Eq (6) is used to obtain the probability distribution of the satisfaction degree *S*_*e*_ in the corresponding OTC drug category (*C*_*i*_) with the influencing factor (*F*_*k*_) as *S*_*e*_, which is shown in Fig 9.

For different categories of drugs, the probability distribution function of the different satisfaction scales in each factor can be determined by Eq (7). To better show, the tonics category (*C*_1_) is used as an example, and the probability distribution of its two influencing factors (*F*_1_ and *F*_2_) is obtained, which are shown as follows:

$$F_{11}(e)=\{\begin{array}{ll} 0, & e\leq 1\\ 0.0986, & 1\leq e<2\\ 0.2821, & 2\leq e<3\\ 1, & e\geq 3 \end{array}$$ (18)

$$F_{12}(e)=\{\begin{array}{ll} 0, & e\leq 1\\ 0.0850, & 1\leq e<2\\ 0.1685, & 2\leq e<3\\ 1, & e\geq 3 \end{array}$$ (19)

Further, the expected vector of the evaluation scale with the factor *F*_*k*_ in different categories of drugs can be obtained by using Eq (8). In this paper, the tonics category (*C*_1_) and the cold and cough category (*C*_2_) are used as examples, whose expectation vectors (***E***_**1**_ and ***E***_**2**_) are respectively as follows:

$$E_{1}=[2.6153,2.7464,2.6852,2.3271,2.3243]$$ (20)

$$E_{2}=[2.6595,2.2752,2.6783,2.3836,2.5383]$$ (21)

Next, according to the Stochastic Dominance Rules, the stochastic dominance relationship between two factors is judged, and the Stochastic Dominance Relationship Matrix between different types of drug satisfaction factors is constructed. *C*_1_ and *C*_2_ are used as examples in this paper, the Stochastic Dominant Relationship Matrix (***R***) between two factors are obtained respectively as follows:

$$R^{1}=[\begin{array}{ccccc} - & - & - & FSD & FSD\\ FSD & - & SSD & FSD & FSD\\ FSD & - & - & FSD & FSD\\ - & - & - & - & SSD\\ - & - & - & - & - \end{array}]$$ (22)

$$R^{2}=[\begin{array}{ccccc} - & FSD & - & FSD & FSD\\ - & - & - & - & -\\ - & FSD & - & FSD & FSD\\ - & FSD & - & - & -\\ - & FSD & - & FSD & - \end{array}]$$ (23)

According to the matrix ***R***, the Stochastic Dominant Relationship Matrix (***RD***) can be obtained. Based on Eqs (22) and (23), the overall priority degree between the two schemes should be calculated through Eq (13). For the factors affecting consumer satisfaction in each category of drugs, the overall priority matrix was constructed. Taking *C*_1_ and *C*_2_ as examples, the corresponding random dominance matrix is given as follows:

$$RD_{1}=[\begin{array}{ccccc} - & 0 & 0 & 1 & 1\\ 0.5947 & - & 0.0061 & 1 & 1\\ 0.3170 & 0 & - & 0 & 0\\ 0 & 0 & 0 & - & 0.0126\\ 0 & 0 & 0 & 0 & - \end{array}]$$ (24)

$$RD_{2}=[\begin{array}{ccccc} - & 1 & 0 & 1 & 0.9968\\ 0 & - & 0 & 0 & 0\\ 0 & 1 & - & 1 & 1\\ 0 & 0.8911 & 0 & - & 0\\ 0 & 0 & 0 & 1 & - \end{array}]$$ (25)

Through the PROMETHEE-II method and the above calculation results, the “*outflow*” and “*inflow*” values between different influencing factors can be obtained by using Eqs (15) and (16). The corresponding ranking values are calculated from Eq (17), which are shown in detail in Table 6.

**Table 6:** The “*outflow*” and “*inflow*” values and the corresponding ranking values between the influencing factors of different categories of OTC drugs.

*C*	*S*_e_	*F*_1_	*F*_2_	*F*_3_	*F*_4_	*F*_5_
*C*_1_	$\Phi_{1}^{+}$	2.0000	2.6009	2.3170	0.0126	0.0000
	$\Phi_{1}^{-}$	0.9117	0.0000	0.0061	3.0000	3.0126
	*Φ*_1_	1.0883	2.6009	2.3108	-2.9874	-3.0126
*C*_2_	$\Phi_{2}^{+}$	2.9968	0.0000	3.0000	0.8911	1.0000
	$\Phi_{2}^{-}$	0.0000	2.8911	0.0000	3.0000	1.9968
	*Φ*_2_	2.9968	-2.8911	3.0000	-2.1089	-0.9968
*C*_3_	$\Phi_{3}^{+}$	3.0000	0.0000	3.0000	0.0000	2.0000
	$\Phi_{3}^{-}$	0.0000	3.0000	0.0000	3.0000	2.0000
	*Φ*_3_	3.0000	-3.0000	3.0000	-3.0000	0.0000
*C*_4_	$\Phi_{4}^{+}$	2.1724	1.9588	1.0000	2.7069	0.0000
	$\Phi_{4}^{-}$	0.2673	0.6120	2.9588	0.0000	4.0000
	*Φ*_4_	1.9051	1.3468	-1.9588	2.7069	-4.0000
*C*_5_	$\Phi_{5}^{+}$	2.8453	1.7479	1.0000	2.0380	0.0000
	$\Phi_{5}^{-}$	0.0000	0.6403	2.6408	0.3501	4.0000
	*Φ*_5_	2.8453	1.1076	-1.6408	1.6879	-4.0000
*C*_6_	$\Phi_{6}^{+}$	1.0000	0.0000	4.0000	0.0879	2.0000
	$\Phi_{6}^{-}$	2.0879	3.0000	0.0000	1.0000	1.0000
	*Φ*_6_	-1.0879	-3.0000	4.0000	-0.9121	1.0000

Therefore, it is easy to know the ranking of the satisfaction factors of each category of OTC drugs, which are shown in Table 7.

**Table 7:** Ranking flow of influencing factors of different categories of OTC drugs.

Drug Category	Category name	Ranking situation graph	Instructions
*C*_1_	Tonics	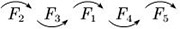	In the column of “Ranking situation graph”, the most important factor affecting customer satisfaction is represented by *F*_i_ on the far left, and the least important factor is on the far right. Along the direction of the arrow, the importance of *F*_*i*_ gradually decreases; The “~” indicates that the importance of the two influencing factors is roughly the same.
*C*_2_	Anti-cold drugs	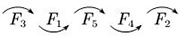	
*C*_3_	Rheumatism and orthopaedic drugs	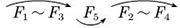	
*C*_4_	Skin drugs	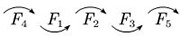	
*C*_5_	Gastrointestinal drugs	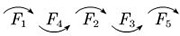	
*C*_6_	Vitamins and calcium	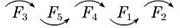	

**Fig 10:**
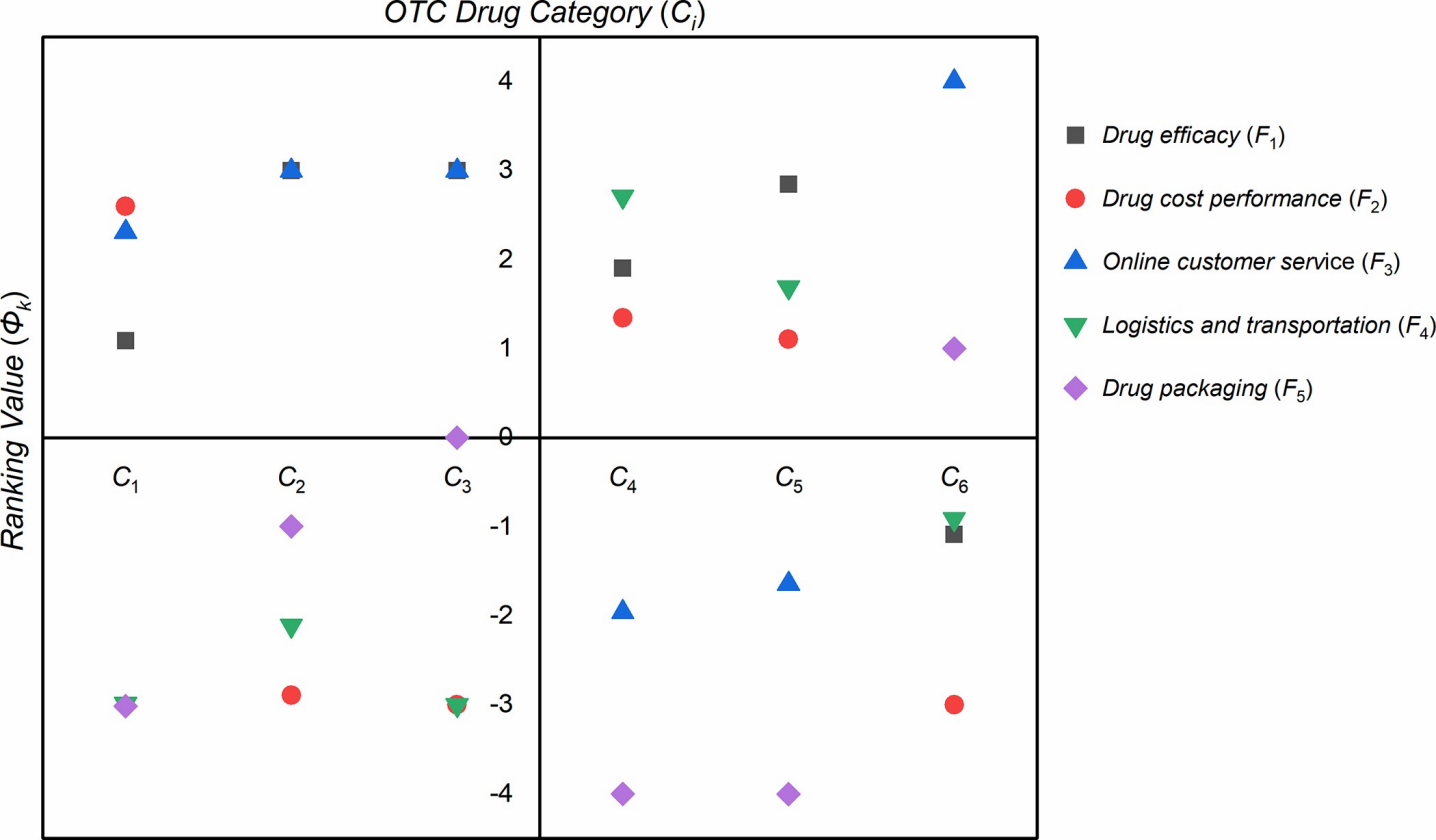
Distribution of ranking values of each factor in different categories of OTC drugs.

To show the results in Table 6 more intuitively, the distribution of ranking values of each factor in different types of drugs is gathered on the same plane, which are shown in Fig 10.

It is easy to know from Fig 10 that for each type of OTC drug sold online, the ranking of factors affecting customer satisfaction is different. Based on the results of Fig 10, the detailed analysis and discussion will be presented below.

## 4 Discussion

Results suggest that for tonic drugs (*C*_1_), “drug cost performance” is judged as the most important factor for customers, and “online customer service” is considered a secondary factor. With the continuous improvement of social living standards and citizens’ health awareness, the public pays more and more attention to health preservation, which makes tonic drugs more and more popular. Due to the wide variety of such drugs, “drug cost performance” has become the primary consideration factor for customers to purchase drugs. Moreover, consumers also pay more attention to customer service in order to obtain targeted consulting results. Because tonic drugs depend on long-term use and their efficacy is slow, customers do not pay much attention to “drug efficacy”. The importance of “drug packaging” and “logistics and transportation” are in the bottom echelon, and these factors have little effect on customer satisfaction.

For anti-cold drugs (*C*_2_), “drug efficacy” and “online customer service” are the most important factors for customers. This is because customers urgently need professional and targeted customer service guidance when they have a cold so that they can purchase drugs quickly; At the same time, they expect the best effect of drugs to relieve their cold symptoms as soon as possible. In contrast, customers place a general emphasis on “drug packaging”. In addition, the “logistics and transportation” factor has not received much attention from customers, this is because when purchasing such drugs, they tend to choose stores that are closer to each other, and the timeliness of drug delivery is inherently high, so they can receive the drugs as soon as possible to relieve cold symptoms. Therefore, customers rarely comment on logistics. It is worth noting that the “drug cost performance” factor ranks last. This is because customers are eager to relieve their cold symptoms, and they will try their best to choose drugs with better efficacy under the advice of customer service, but such drugs are not necessarily cost-effective.

For rheumatology and orthopedics drugs (*C*_3_), “drug efficacy” and “online customer service” are the two most important factors. Obviously, in the early stage of the disease, customers want to choose better drugs with the help of customer service to relieve their injuries, and they don’t care much about packaging. In fact, since rheumatism is a chronic autoimmune disease, most patients will go to the hospital for regular review and treatment. At the same time, patients usually go to the hospital for diagnosis and treatment as soon as possible after injury, rather than buying drugs online first. Coincidentally, when crawling the relevant data, this study found that the sales and review data of this type of drug are the least among the six types of OTC drugs. This perfectly confirms the above conclusion.

For skin drugs (*C*_4_), because customers are eager to relieve symptoms such as skin infections, the factors they value more are “logistics and transportation”, followed by “drug efficacy”. The importance of “drug cost performance” and “online customer service” ranked in the middle. Some patients have used certain drugs (such as loratadine tablets, compound dexamethasone acetate cream, etc.) many times, therefore, they identify a certain drug as a commonly used drug. The “packaging” factor pales in importance compared to other factors.

For gastrointestinal drugs (*C*_5_), expecting good relief of gastrointestinal discomfort, the most favored factor for customers is “drug efficacy”, followed by “logistics and transportation”. However, they pay less attention to the “drug cost performance”, and pay the least attention to the packaging of drugs. According to some reviews, most customers buy a drug multiple times on their own, so the “online customer service” factor is less important.

For vitamins and calcium (*C*_6_), the most important factor is “online customer service”. At this stage, with the increase of work and study pressure, the public has gradually built up an awareness of improving immunity and began to consciously buy vitamin calcium health care drugs. Customers expect more professional customer service guidance when purchasing, and they pay more attention to customer service. The “packaging” factor comes in second because most vitamin calcium drugs are bottled, according to the relevant reviews, customers pay more attention to the packaging after receiving the goods, such as whether the bottle is compressed. Due to the long-term use of such drugs, their logistics and drug efficacy have received less attention. In addition, because the prices of these drugs are generally high, the “drug cost performance” factor is the least important.

Since the drug is a special commodity, their safety and effectiveness is a special concern. While providing convenience to many pharmaceutical consumers, Alibaba Health Pharmacy should pay attention to the safety and effectiveness of drugs and strive to serve consumers. On the one hand, customer satisfaction should be continuously improved; on the other hand, the healthy development of pharmaceutical e-commerce should be paid more attention to. Combined with the previous discussion, this paper puts forward the following suggestions for the pharmaceutical e-commerce of Ali Health Pharmacy:

For tonic drugs, efforts should be made to improve to remove a batch of drugs with poor customer satisfaction. For those with high customer satisfaction, their cost performance needs to be continuously improved, and signature drugs should be built and maintained. For cold and cough drugs, as well as gastrointestinal drugs, pharmaceutical e-commerce companies should pay more attention to efficacy, and put more drugs with quick effects on the shelves to solve the urgent needs of customers; at the same time, the professionalism of the online customer service level should be improved so that they can be provided with more accurate drug purchase guidance. For rheumatic orthopedics drugs, the professional level of customer service should also be continuously improved to guide consumers to make drug purchase choices suitable for their own conditions. For skin drugs, e-commerce companies should pay more attention to logistics and transportation services, and choose a group of courier companies with high efficiency and fast delivery time to carry out cooperation. For Vitamin Calcium Health Care drugs, pharmaceutical e-commerce should focus on online customer service. When guiding customers to purchase drugs, customer service should ask customers about various aspects of the situation. For example, some people with hypercalcemia, hyperuricemia, calcium-containing kidney stones or a history of kidney stones should not use a certain drug. For a similar situation in the above example, customer service personnel must provide consumers with a full range of drug purchase guidance.

The study has some limitations. As time goes by, the online review information of drugs will be expanded by a large amount. The existing LDA theme model may have a problem, that is, the degree of correlation between the relevance and contribution of the variables may become blurred, which reduces the accuracy of the theme extraction results. With more and more network words, a large number of new words are emerging, resulting in more words being given more complex emotional colors, which brings more challenges to the emotional analysis. How to deal with these challenges is the direction of further optimization of this study in the future. In addition, perhaps the data collected in this study is not yet extensive, making some controversy in the conclusions of the study possible. In our future studies, we will consider continuing to collect more data and conducting replicated analyses on a wider range of different OTC drugs sold online to make the findings more accurate and scientific.

## 5 Conclusion

This paper proposes a method for evaluating customer satisfaction with online drug sales based on online text evaluation information and conducts an empirical study. The research in this paper is the first time to introduce online review information into the field of pharmaceutical e-commerce to explore customer satisfaction, which is cutting-edge and representative. Compared with previous research, it also has the following highlights:

Firstly, when determining the factors affecting customer satisfaction, methods such as literature research and expert interviews are not used because they are highly subjective. Instead, it uses the online reviews of a large number of customers to mine potential topics through the LDA topic model. The number of topics is accurately determined by using topic-perplexity, topic-coherence, *pyLDAvis* visualization and other methods, which greatly reduces the degree of subjectivity. Secondly, Sentiment Analysis technology is utilized, and some popular terms have been integrated into the sentiment dictionary. This paper successfully quantifies the sentiment tendency of each review, which provides solid support for in-depth research. Thirdly, this paper uses the Stochastic Dominance Rules to evaluate the stochastic dominance relationship between two factors. Using the characteristics of this criterion, that is, it can assist factor ranking work without strict assumptions, which further enhances the credibility and practical significance of the results. Finally, this paper uses the PROMETHEE-Ⅱ method to rank each factor with the help of the stability of its mathematical properties, which clearly and intuitively shows the arrangement of the importance of different factors. Its use in conjunction with the Stochastic Dominance Rules played a key role in this study.

The method proposed in this paper has certain academic value, and provides research ideas for the evaluation of customer satisfaction with drugs in other online pharmacies. It also provides a reasonable strategy for medical e-commerce, which is of great significance. In addition, this study was conducted only for over-the-counter medicines from AliHealth Pharmacy. In the future, we intend to conduct further similar studies on other online pharmacies. We will compare these results longitudinally to draw more insightful conclusions.

## Supporting information

S1 Dataset Sample data set. (TXT)

## References

[1] Feng J, Yao Z. Consumer-Generated Reviews Based on Social Learning Theory. lmplications for Purchase Decision. Chinese Journal of Management Science. 2016; 24(9), 106–114.

[2] Chevalier J A, Mayzlin D. The effect of word of mouth on sales: Online book reviews[J]. Journal of marketing research. 2006; 43(3): 345–354. https://doi.org/10.1509%2Fjmkr.43.3.345.

[3] Lee Y J, Hosanagar K, Tan Y. Do I follow my friends or the crowd? Information cascades in online movie ratings. Management Science. 2015; 61(9): 2241–2258. 10.1287/mnsc.2014.2082.

[4] Liao H C, Liu F, Lu K Y, Zhu T, Luo L. Online Medical Reviews on Patient Behavior Mining and Its Applications in Medical Decision-Making and Management. Journal of University of Electronic Science and Technology of China (Social Sciences Edition). 2022; 24(3), 1–22.

[5] Wu X, Liao H. Modeling personalized cognition of customers in online shopping. Omega. 2021; 104: 102471. 10.1016/j.omega.2021.102471.

[6] Gao H M, Liu H W, Zhan M J, Fan M T, Liang Z Y. Research on the Impact of Online Reviews and Product Involvement on Virtual Shopping-Cart Decision-making Based on Consumer Involvement. Chinese Journal of Management Science. 2021; 29(6), 211–222.

[7] Miao X M, Chen Y T, Min C M. Study on Consumer Satisfaction of Tangshan Hot Springs based on ISM and Online Reviews. Chinese Journal of Management Science. 2019; 27(7), 186–194.

[8] Chatterjee S, Mandal P. Traveler preferences from online reviews: Role of travel goals, class and culture. Tourism Management. 2020; 80, 104108. 10.1016/j.tourman.2020.104108.

[9] Zhao M, Shen X, Liao H, Cai M. Selecting products through text reviews: An MCDM method incorporating personalized heuristic judgments in the prospect theory. Fuzzy Optimization and Decision Making. 2022; 21(1), 21–44. 10.1007/s10700-021-09359-8.

[10] Battineni G., Baldoni S., Chintalapudi N., Sagaro G. G., Pallotta G., Nittari G., et al. Factors affecting the quality and reliability of online health information. 2020; Digital Health, 6, 2055207620948996. doi: 10.1177/2055207620948996 32944269 PMC7466903

[11] Nittari G., Savva D., Tomassoni D., Tayebati S. K., & Amenta F. Telemedicine in the COVID-19 Era: A Narrative Review Based on Current Evidence. International Journal of Environmental Research and Public Health. 2022; 19(9), 5101. doi: 10.3390/ijerph19095101 35564494 PMC9105428

[12] Baldoni S., Pallotta G., Traini E., Sagaro G. G., Nittari G., & Amenta F. A survey on feasibility of telehealth services among young Italian pharmacists. Pharmacy Practice (Granada). 2020; 18(3). doi: 10.18549/PharmPract.2020.3.1926 32802217 PMC7416313

[13] Zhang C X, Zhao M, Cai M Y, Xiao Q R. Multi-stage multi-attribute decision making method based on online reviews for hotel selection considering the aspirations with different development speeds. Computers & Industrial Engineering. 2020; 143, 106421. 10.1016/j.cie.2020.106421.

[14] Najmi E, Hashmi K, Malik Z, Rezgui A, Khan H U. CAPRA: a comprehensive approach to product ranking using customer reviews. Computing. 2015; 97(8), 843–867. 10.1007/s00607-015-0439-8.

[15] Liu Y, Bi J W, Fan Z P. Ranking products through online reviews: A method based on sentiment analysis technique and intuitionistic fuzzy set theory. Information Fusion. 2017; 36, 149–161. 10.1016/j.inffus.2016.11.012.

[16] Yang X, Yang G, Wu J. Integrating rich and heterogeneous information to design a ranking system for multiple products. Decision Support Systems. 2016; 84, 117–133. 10.1016/j.dss.2016.02.009.

[17] Li Y, Wu C, Luo P. Rating online commodities by considering consumers’ purchasing networks. Management Decision. 2014; 52(10), 2002–2020. 10.1108/MD-04-2014-0188.

[18] Lin S S, Yu G F. Product Selection Methods Based on Prospect Theory and Online Reviews. Operations Research and Management Science. 2021; 30(2), 191–195.

[19] You T H, Zhang J, Fan Z P. Method for Selecting Desirable Product (s) Based on Online Rating Information and Customer’s Aspirations. Chinese Journal of Management Science. 2017; 25(11), 94–102.

[20] Imbergamo C, Brzezinski A, Patankar A, Weintraub M, Mazzaferro N, Kayiaros S. Negative Online Ratings of Joint Replacement Surgeons: An Analysis of 6,402 Reviews. Arthroplasty Today. 2021; 9(7), 106–111. 10.1016/j.artd.2021.05.005. 34189214 PMC8217335

[21] Jung Y, Suh Y. Mining the voice of employees: A text mining approach to identifying and analyzing job satisfaction factors from online employee reviews. Decision Support Systems. 2019; 123, 113074. 10.1016/j.dss.2019.113074.

[22] Huang Z, Li H. The factors influencing online reviews of online pharmacies based on big data. Journal of Shenyang Pharmaceutical University. 2016; 33(10), 833–838.

[23] Guo Y, Barnes S J, Jia Q. Mining meaning from online ratings and reviews: Tourist satisfaction analysis using latent dirichlet allocation. Tourism management. 2017; 59, 467–483. 10.1016/j.tourman.2016.09.009.

[24] Tirunillai S, Tellis G J. Mining marketing meaning from online chatter: Strategic brand analysis of big data using latent dirichlet allocation. Journal of marketing research. 2014; 51(4), 463–479. https://doi.org/10.1509%2Fjmr.12.0106.

[25] Jung Y, Suh Y. Mining the voice of employees: A text mining approach to identifying and analyzing job satisfaction factors from online employee reviews. Decision Support Systems. 2019; 123, 113074. 10.1016/j.dss.2019.113074.

[26] Zhong J J, Liu W, Wang S L, Yang H. Review of Methods and Applications of Text Sentiment Analysis. Data Analysis and Knowledge Discovery. 2021; 5(6), 1–13.

[27] Srinivas S, Rajendran S. Topic-based knowledge mining of online student reviews for strategic planning in universities. Computers & Industrial Engineering. 2019; 128, 974–984. 10.1016/j.cie.2018.06.034.

[28] Zhang X, Fan Z P. A Method for Large Group Decision Making with Multi-attribute and Multi-identifier Based on Stochastic Dominance Rules. Systems Engineering. 2010; 28(2), 24–29.

[29] Nowak M. Preference and veto thresholds in multicriteria analysis based on stochastic dominance. European Journal of Operational Research. 2004; 158(2), 339–350. 10.1016/j.ejor.2003.06.008.

[30] Martel J M, Zaras K. Stochastic dominance in multicriterion analysis under risk. Theory and Decision. 1995; 39(1), 31–49. 10.1007/BF01078868.

[31] Zaras K. Rough approximation of a preference relation by a multi-attribute dominance for deterministic, stochastic and fuzzy decision problems. European Journal of Operational Research. 2004; 159(1), 196–206. 10.1016/S0377-2217(03)00391-6.

[32] Brans J P, Vincke P. Note—A Preference Ranking Organisation Method: (The PROMETHEE Method for Multiple Criteria Decision-Making). Management science. 1985; 31(6), 647–656. 10.1287/mnsc.31.6.647.

[33] Zhang Y, Fan Z P, Liu Y. A method based on stochastic dominance degrees for stochastic multiple criteria decision making. Computers & Industrial Engineering. 2010; 58(4), 544–552. 10.1016/j.cie.2009.12.001.

[34] Blei D M, Ng A Y, Jordan M I. Latent dirichlet allocation. Journal of machine Learning research. 2003; 3(1), 993–1022.

[35] Han Y N, Liu J W, Luo X L. A Survey on Probabilistic Topic Model. Chinese Journal of Computers. 2021; 44(06): 1095–1139.

[36] Feng K, Yang Q, Chang X Y, Li Y L. Customer Satisfaction Evaluation Method for Fresh E-commerce Based on Online Reviews and Stochastic Dominance Rules. Chinese Journal of Management Science. 2021; 29(2), 205–216.

[37] Wang T, Yang W Z. Review of Text Sentiment Analysis Methods. Computer Engineering and Applications. 20221; 57(12), 11–24.

[38] Liang X, Jiang Y P, Gao M. Product Selection Methods Based on Online Reviews. Journal of Northeastern University (Natural Science). 2017; 38(1), 143–147.

[39] Blei D, Ng A, Jordan M. Latent dirichlet allocation. Advances in neural information processing systems. 2001; 14.

[40] Amoualian H, Lu W, Gaussier E, Balikas G, Amini M R, Clausel M. Topical coherence in LDA-based models through induced segmentation[C]// Proceedings of the 55th Annual Meeting of the Association for Computational Linguistics (Volume 1: Long Papers). 2017: 1799–1809.

[41] Mimno D, Wallach H, Talley E, Leenders M, McCallum A. Optimizing semantic coherence in topic models[C]// Proceedings of the 2011 conference on empirical methods in natural language processing. 2011: 262–272.

[42] Newman D, Lau J H, Grieser K, Baldwin T. Automatic evaluation of topic coherence[C]// Human language technologies: The 2010 annual conference of the North American chapter of the association for computational linguistics. 2010: 100–108.

[43] Lau J H, Baldwin T. The sensitivity of topic coherence evaluation to topic cardinality[C]// Proceedings of the 2016 conference of the North American chapter of the Association for Computational Linguistics: Human language technologies. 2016: 483–487.

